# The Genetic Basis of Probable REM Sleep Behavior Disorder in Parkinson’s Disease

**DOI:** 10.3390/brainsci13081146

**Published:** 2023-07-30

**Authors:** Santiago Perez-Lloret, Guenson Chevalier, Sofia Bordet, Hanny Barbar, Francisco Capani, Lucas Udovin, Matilde Otero-Losada

**Affiliations:** 1 Observatorio de Salud Pública, Vicerrectorado de Investigación e Innovación Académica, Pontificia Universidad Católica Argentina (UCA), Consejo Nacional de Investigaciones Científicas y Técnicas (UCA-CONICET), Buenos Aires C1107AAZ, Argentina; 2 Centro de Investigaciones en Psicología y Psicopedagogía (CIPP), Facultad de Psicología y Psicopedagogía, Pontificia Universidad Católica Argentina (UCA), Buenos Aires C1107AFB, Argentina; sofia.bordet@gmail.com; 3 Departamento de Fisiología, Facultad de Medicina, Universidad de Buenos Aires (UBA), Buenos Aires C1053ABH, Argentina; 4 Centro de Altos Estudios en Ciencias Humanas y de la Salud, Universidad Abierta Interamericana, Consejo Nacional de Investigaciones Científicas y Técnicas, CAECIHS.UAI-CONICET, Buenos Aires C1270AAH, Argentina; guensonchevalier@yahoo.com (G.C.); hannyjrb@gmail.com (H.B.); franciscocapani@hotmail.com (F.C.); lucas2304@hotmail.com (L.U.); molly1063@gmail.com (M.O.-L.); 5 Instituto de Ciencias Biomédicas, Facultad de Ciencias de la Salud, Universidad Autónoma de Chile, Santiago 7500912, Chile

**Keywords:** single nucleotide polymorphism, Parkinson’s disease, REM-sleep behavior disorders, pathophysiology, PD-related variants

## Abstract

Patients with Parkinson’s Disease (PD) experience REM sleep behavior disorder (RBD) more frequently than healthy controls. RBD is associated with torpid disease evolution. To test the hypothesis that differential genetic signatures might contribute to the torpid disease evolution in PD patients with RBD we compared the rate of genetic mutations in PD patients with or without probable RBD. Patients with a clinical diagnosis of PD in the Parkinson’s Progression Markers Initiative (PPMI) database entered the study. We excluded those with missing data, dementia, psychiatric conditions, or a diagnosis change over the first five years from the initial PD diagnosis. Probable RBD (pRBD) was confirmed by a REM Sleep Behavior Disorder Screening Questionnaire score > 5 points. Logistic regression and Machine Learning (ML) algorithms were used to relate Single Nucleotide Polymorphism (SNPs) in PD-related genes with pRBD. We included 330 PD patients fulfilling all inclusion and exclusion criteria. The final logistic multivariate model revealed that the following SNPs increased the risk of pRBD: GBA_N370S_rs76763715 (OR, 95% CI: 3.38, 1.45–7.93), SNCA_A53T_rs104893877 (8.21, 2.26–36.34), ANK2. CAMK2D_rs78738012 (2.12, 1.08–4.10), and ZNF184_rs9468199 (1.89, 1.08–3.33). Conversely, SNP COQ7. SYT17_rs11343 reduced pRBD risk (0.36, 0.15–0.78). The ML algorithms led to similar results. The predictive models were highly specific (95–99%) but lacked sensitivity (9–39%). We found a distinctive genetic signature for pRBD in PD. The high specificity and low sensitivity of the predictive models suggest that genetic mutations are necessary but not sufficient to develop pRBD in PD. Additional investigations are needed.

## 1. Introduction

Parkinson’s is the second most frequent neurodegenerative disorder worldwide [1]. Patients are affected by motor and non-motor symptoms, with the latter being the most disturbing [2]. Its main motor symptoms are bradykinesia, resting tremor, rigidity, and postural abnormalities [3]. Patients are also affected by non-motor symptoms, including mood disorders, trouble sleeping, dysautonomia, cognitive dysfunction, and pain, among others [3]. Sleep disorders affect PD patients frequently and negatively impact on quality of life [4,5]. Insufficient sleep interferes with routine activities and can also aggravate motor symptoms in PD. Some of the most frequent disorders are REM sleep behavior disorder (RBD), insomnia, restless legs syndrome (RLS) and periodic limb movement disorder, circadian rhythm sleep disorders, nocturia, sleep-disordered breathing (SBD), and excessive daytime sleepiness.

Compared with the general population, RBD is more prevalent in PD patients, affecting between 20% and 75% of patients [6,7,8,9]. Furthermore, RBD often precedes the onset of motor symptoms in PD by several years and can serve as an early marker or prodromal disease symptom [10]. However, RBD may also develop after PD onset [11].

PD can be sporadic or familial when autosomal mutations are present [12,13]. However, recent evidence indicates that genetic mutations also contribute in non-negligible ways to sporadic PD [14]. A recent meta-analysis of genome-wide association studies (GWAS) included the analysis of 7.8 M SNPs in 37.7 K cases, 18.6 K UK Biobank proxy-cases (having a first-degree relative with PD), and 1.4 M controls [14]. The authors could identify 90 variants that explained 16–36% of the heritable risk of PD depending on prevalence. Interestingly, the presence of some mutations determines distinct phenotypes. For example, in PD patients with mutations in the Leucine-rich repeat kinase 2 (LRRK2) gene, the disease progresses slower than those without mutations and are less frequently affected by non-motor symptoms, including olfaction, REM-sleep behavior disorders, and cognitive dysfunction [15]. PD may also relate to mutations in the GBA gene, encoding the lysosomal enzyme glucocerebrosidase (Gcase) [14]. A recent meta-analysis has shown that PD patients with *GBA* mutations suffer from accelerated progression of the disease, with more frequent motor fluctuations, depression, and dementia compared to non-affected patients [16].

RBD is a sleep disorder characterized by the loss of muscle atonia during rapid eye movement (REM) sleep, resulting in individuals physically acting out their dreams [17,18]. In individuals with RBD, muscle atonia typical of REM sleep is incomplete or gone, leading to dream-enactment behaviors. These range from mild—simple limb twitches or gestures—to more vigorous and potentially harmful actions like kicking, punching, or even jumping out of bed [17,18]. RBD has been linked to neurodegenerative disorders and synucleinopathies, in particular, Parkinson’s disease (PD), multiple system atrophy, and dementia with Lewy bodies [19].

A disruption in the normal inhibition of muscle activity during REM sleep causes RBD [20,21,22,23]. Individuals with RBD fail at maintaining the typical muscle atonia during REM sleep and then enact dream-related behaviors. The pontomedullary reticular formation, including the subcoeruleus nucleus and other brainstem regions, plays a crucial role in generating REM sleep atonia [23]. Dysfunction or degeneration of these regions disrupts the inhibitory signals sent to the spinal motor neurons, disinhibiting motor activity during REM sleep.

The genetics of idiopathic RBD have been widely studied. In a large genome-wide association study in RBD patients, six RBD-associated loci in five genomic regions were identified: two loci near SCARB2 and INPP5F and three previously reported loci near SNCA, GBA, and TMEM175 [24]. In another case-control study involving 1072 idiopathic RBD patients and 9505 controls, Single Nucleotide Polymorphisms (SNPs) in the HLA-DRB1 were the only genetic factors predicting RBD [25]. SNPs in BST1 and LAMP3 have also been connected with RBD in a study involving 1039 patients and 1852 controls [26]. GBA variants also robustly and differentially increase the risk of idiopathic RBD in a study involving 4147 individuals [27]. The involvement of GBA variants was also associated with RBD in other studies [28,29]. Carriers of the LRRK2 p.N551K-p.R1398H-p.K1423K haplotype have a reduced risk of developing RBD [30]. Conversely, SNPs in the NPC1, which encodes a lysosomal protein involved in cholesterol transport [31], and in the sphingomyelin phosphodiesterase 1 (SMPD1) gene did not show any association with idiopathic RBD [32]. PD-related variants in LRRK2 were also not related to RBD [33,34,35].

Patients who develop PD usually have a more malignant phenotype with a faster rate of disease progression [11]. Indeed, PD patients with RBD (PD-RBD) display higher UPDRS-III motor scores and Hoehn–Yahr scores [9,36], depression, anxiety, apathy, fatigue [11], sleepiness, and cognitive impairment [28].

The reasons for the worst outcome in PD-RBD are currently unknown. We hypothesized that a differential genetic signature might contribute to the torpid evolution of the disease. Therefore, we set out to compare the rate of genetic mutations in patients with PD and probable RBD vs. patients without RBD.

## 2. Materials and Methods

### 2.1. Participants

The Parkinson’s Progression Markers Initiative (PPMI) is an ongoing multicenter observational study focused on identifying disease biomarkers in PD patients attending clinical centers from all over the world [37]. The overall goal of PPMI is to identify markers of disease progression to accelerate therapeutic trials to reduce progression of PD disability. All participants signed written informed consent, and the review board of each center approved the protocol. Information is de-identified and shared with involved and uninvolved investigators. We extracted information only from each participant’s baseline visit.

For our study, we selected patients with a clinical diagnosis of idiopathic PD based on the UKPDBBS or the MDS criteria. Patients with missing data, demented patients, those whose PD diagnosis was changed during the first five years after the diagnosis of PD, or those with psychiatric conditions, were excluded.

### 2.2. Assessment of Probable REM Sleep Behavior Disorder

The “REM Sleep Behavior Disorder Screening Questionnaire” (RBDSQ) was used to screen RBD symptoms [38]. The RBDSQ is a 10-item patient self-rating questionnaire (maximum total score of 13 points) covering the clinical features of RBD, including dream content, nocturnal movements, injuries to self or bed partner, types of motor behaviors during the night, nocturnal awakenings, sleep disruption, and the presence of neurological diseases. The scale was originally developed in English and German, but it has been translated into other languages [39]. Administration time is typically 5 min.

Sensitivity and specificity for polysomnographic-confirmed RBD are 0.96 and 0.56, respectively, considering an RBDSQ score of five points as a positive result [38]. Therefore, we classified PD patients as probable RBD (pRBD) if the RBDSQ score was above 5 points.

### 2.3. Genomic Data Processing

As part of the screening or baseline visit, blood was drawn, and whole-genome sequencing was performed using a Macrogen Inc. (Seoul, South Korea) sequencer on whole blood-extracted DNA samples [37]. One microgram of each DNA sample was fragmented with Covaris System and prepared following the Illumina TruSeq DNA Sample preparation guide to obtain a final library of 300–400 bp average insert size. Libraries were multiplexed and sequenced on the Illumina HiSeq X platform. Paired-end read sequences were aligned to the GRCh37-hs37d5 genome using the Burrows–Wheeler aligner-maximal exact matches algorithm (BWA-MEM v0.7.13). The Bamsormadup2 tool (v2.0.87) was used to filter duplicates and sort aligned bam files. After filtering duplicated read sequences, the reads were realigned and recalibrated using the GATK pipeline (v3.5). Haplotype caller in the GATK pipeline was used to call variants, including single nucleotide variants (SNVs) and small In/Dels, and to generate genome VCFs. Using the hg38 aligned cohort VCF files from the whole-genome sequencing data, genotype information was extracted using BCF tools and PLINK. We considered the alleles of the 72 variants available in the PPMI database that are associated with an increased PD risk, as identified in a recent large case-control study [14]. We focused on SNPs with a minimum call rate of 95%, a minor allele frequency (MAF) > 1%, and Hardy–Weinberg equilibrium *p*-values > 0.05.

### 2.4. Statistical Analysis

Numerical variables were expressed as means ± standard deviation and the categorical ones in percentages. Differences between PD patients with and without NOH were analyzed with a T-test or Chi-square test. We used a logistic regression model to identify SNPs independently associated with probable RBD. We used the Akaike Information Component (AIC) to determine the genetic model that best fitted the data. In the “dominant” model, having one copy of the SNP modified the risk of the outcome. According to the “recessive” model, the risk of the outcome was only modified by the presence of the SNP in both alleles. In the “additive” model, having one or two copies of the SNP affected differently the risk of the outcome. For the multivariate model, we selected the one with the highest AIC. The Benjamini and Hochberg step-up procedure was used to control the False Discovery Rate and adjust the *p*-values. All analyses were performed using R Statistical Software (v4.1.2; R Core Team 2021).

### 2.5. Machine-Learning Models

We used Machine-Learning (ML) algorithms to further model the relationship between probable RBD with SNPs and other covariates. We fitted Logistic regression, Bayes Naïve, Decision trees, Boosted Decision trees, Neural Networks, Support-Vector Machines (SVM), and Random Forest models to a “development” and “validation” subsamples comprising 70%/30% of the original sample. For each model, we computed the Area Under the Curve of the Receiver–Operator Curve (AUC-ROC) and the sensitivity and specificity for detecting pRBD. In the second row of analyses, we included clinical covariates in the models. We also used Leave-one-out CV (LOOCV) as the resampling technique, aiming at maximizing the size of the development sub-sample. In this cross-validation technique, ML models are fitted to the whole sample minus 1 participant, who serves to test the model validity. This procedure is iterated through the whole sample. Therefore, both the development and validation subsamples included all participants. All analyses were performed with R, using the following libraries: rstatix, e1071, C50, NeuralNet, Kernlab, RandomForest, and caret.

## 3. Results

### 3.1. Characteristics of the Sample

Six hundred and seventy-four patients fulfilled the inclusion criteria. The final sample included 330 PD patients, as 344 had missing data. Characteristics of patients with pRBD or no RBD are shown in Table 1. Most notably, patients with pRBD had a younger disease onset, a longer disease duration, more severe motor symptoms, more frequent antiparkinsonian treatment, and more frequent motor fluctuations.

### 3.2. Genetic Factors Connected with RBD

We observed significant differences between PD patients with pRBD and without RBD in 11 SNPs (Table 2). The final multivariate model revealed that the SNP GBA_N370S_rs76763715, SNCA_A53T_rs104893877, ANK2.CAMK2D_rs78738012, ZNF184_rs9468199 increased the risk of pRBD. Conversely, the SNP COQ7.SYT17_rs11343 reduced pRBD risk.

### 3.3. Machine-Learning Models

We fitted a series of ML models to corroborate these findings. Table 3 shows the performance of ML models in the “validation” subsample, which comprised 1/3 of the original sample. The logistic regression and Bayes Naïve algorithms showed a sensitivity of 39% and a specificity of 95%. The Neural Network model had the highest AUC-ROC; however, sensitivity and specificity were not different from the logistic regression or Bayes Naïve models.

To increase the predictive power of the ML models, we included Levodopa-Equivalent Daily Dose, MDS-UPDRS I + II + III, and Motor Fluctuations, which show differences in patients with pRBD or no RBD (Table 1). Inclusion of Age at Disease Onset and Disease Duration was not possible due to missing data (Table 1). The models’ performance and coefficients for the variables included are shown in Table 4. The most significant change was a 10% increase in sensitivity, which was lost when the LOOCV method was used to build the ML model.

## 4. Discussion

We hypothesized that PD patients with pRBD may have a different genetic background as compared with PD patients without RBD. In this study, we observed that pRBD was directly associated with GBA_N370S_rs76763715, SNCA_A53T_rs104893877, and ANK2.CAMK2D_rs78738012, ZNF184_rs9468199, and indirectly, with SNP COQ7.SYT17_rs11343. These results support our initial hypothesis, shedding light on the possible origin of the worst prognosis observed in patients with RBD.

Before further discussing these findings, the lack of polysomnographic confirmation of RBD diagnosis deserves a few words. Both the absence of muscle atonia during REM sleep and the presence of dream-enactment behaviors during polysomnography are required to confirm RBD diagnosis [40]. The differential diagnosis of RBD includes non-REM parasomnia (confusional arousals, sleepwalking, sleep terrors), nightmares, benign sleeptalking, nocturnal frontal lobe epilepsy, and sleep-fragmenting conditions like obstructive sleep apnea and periodic limb movements, among others [38]. As polysomnography is impractical for large multicentric studies, surveys are commonly used [9]. The RBDSQ used here is highly sensitive but unspecific. A negative result drastically reduced RBD likelihood, while a positive result should be confirmed by PSG, which was not done in this case. However, there is no theoretical background to think that RBD differential diagnoses are related to the SNPs assessed in this study. Therefore, the relationships between RBD and the SNPs observed were probably underestimated due to the presence of non-RBD patients among those with pRBD, and less strong relationships may have been missed out. Further research is needed studying confirmed RBD patients. Finally, we didn’t assess the “codominant” model—when the two alleles are expressed separately, yielding different effects. The database did not include the data needed to assess this model.

Several studies found that GBA and SNCA SNPs are more frequent among patients with idiopathic RBD compared to controls. In line with these findings, we observed that GBA_N370S_rs76763715 and SNCA_A53T_rs104893877 related to more frequent pRBD among people living with PD. Compared with non-carriers, PD patients with SNCA variants have more frequent non-motor symptoms, including RBD, and a similar disease progression rate (SNPs rs2870004, rs356182, rs5019538, and rs763443) [41]. Some GBA variants, like rs2230288/E326K, rs75548401/T369M, and rs369068553/V460L, are also linked to more frequent non-motor symptoms, including RBD, but also to a more aggressive motor disease [42,43,44]. Therefore, the presence of genetic mutations in the SNCA gene may explain the worse prognosis in PD-RBD.

A study of Chinese PD patients showed that the variant rs9468199 in the ZNF184 gene was associated with more frequent RBD [45]. The ZNF184 encodes a Kruppel C2H2-type zinc-finger protein family member, which participates in gene expression regulation. One study found that REM sleep deprivation in rats induced significant ZNF184 down-regulation in the brain [46]. The effects of this mutation on other features of PD are unknown.

We report for the first time the associations between pRBD and ANK2.CAMK2D_rs78738012 and COQ7.SYT17_rs11343 variants. The ANK2 gene encodes a member of the ankyrin family of proteins, linking the integral membrane proteins to the underlying spectrin–actin cytoskeleton. Ankyrins play key roles in organizing the axon initial segment and nodes of Ranvier, and they organize and stabilize neurotransmitter receptors, modulate dendritic spine morphology, and control adhesion to the presynaptic site [47]. The product of the CAMK2 belongs to the serine/threonine protein kinase family and to the Ca (2+)/calmodulin-dependent protein kinase subfamily. Calcium signaling is crucial for several aspects of plasticity at glutamatergic synapses. Interestingly, Camk2b knockout mice show reduced sleep [48]. The phosphorylation states of CaMKIIβ appear to control sleep induction and maintenance processes differently.

The SYT17 encodes the Synaptotagmin 17 protein, which has calcium ion binding activity, phospholipid binding activity, and syntaxin binding activity. It is involved in the positive regulation of dendrite extension. The suprachiasmatic nucleus—the master biological clock of the brain—expresses high levels of SYT17 [49]. Several findings suggest that SYT17 is involved in regulating circadian rhythms. The best-fitting model for the risk of this gene was the “recessive” model, suggesting that the SNPs might be linked to a loss of gene function. Therefore, only when both alleles are compromised protein expression might fall below critical levels. Further research is needed to assess these factors’ importance in RBD pathophysiology and their impact on other features of PD.

Our predictive models were highly specific but poorly sensitive. Namely, PD patients without pathogenic SNPs showed a minimal risk of suffering from RBD. PD patients with pathogenic mutations had a moderate increase in the risk of pRBD. These findings suggest that genetic background is necessary for the development of pRBD but not sufficient. The non-genetic factors that might contribute to the development of idiopathic RBD include smoking, previous head injury, fewer years of formal schooling, and working on a farm [50]. However, the risk factors for pRBD have been less frequently studied. The inclusion of clinical variables like PD severity, levodopa-equivalent daily dose, or the presence of motor fluctuation in the logistic predictive model increased sensitivity by 10%. These factors might contribute to, while others still unknown, are necessary to develop pRBD.

Logistic regression is the cornerstone of multivariate testing and is widely used in epidemiological studies [51,52,53]. The recent development in ML algorithms may provide more sophisticated and powerful methods for detecting associations between variables in epidemiological studies. We compared the results of several ML algorithms with regression logistics in our dataset. More sophisticated algorithms did not outperform logistic regression, emphasizing the power of this old technique.

## 5. Conclusions

We found a distinctive genetic signature for pRBD in PD. SNPs in genes GBA, SNCA, ANK2.CAMK2D and ZNF184 increased while in COQ7.SYT17 reduced the risk of pRBD. Genetic mutations appear necessary but not sufficient to develop RBD. Unaccounted factors remain obscure and additional studies are needed.

## 6. Future Directions

We hypothesized that the more torpid evolution of patients with PD and RBD could result from a genetic mutation. We observed that PD patients with pRBD had an increased frequency of mutations in the GBA and SNCA genes, which relate to a worse prognosis. We also observed mutations in the ANK2.CAMK2D and ZNF184 genes increased the risk of pRBD in PD, while mutations in the COQ7.SYT17 reduced it. Further research is needed to evaluate the pathophysiological implications of these mutations. Furthermore, they might also represent therapeutic targets worth studying.

The main limitation of this study, which should be addressed in future studies, is the inclusion of polysomnography-unconfirmed cases of RBD. The lack of polysomnographic confirmation and the relatively low sample size might have limited our ability to find mutations significantly associated with RBD. In other words, we might have missed some mutations weakly related to RBD. Finally, future studies should address the presence and effects of co-dominant mutations.

## Figures and Tables

**Table 1:** Characteristics of PD patients with probable REM-sleep behavior disorder (pRBD) or no RBD.

Characteristic	Overall (N = 330)	No RBD (N = 240)	pRBD (N = 90)	*p*-Value
Years of education				0.008
Mean (SD)	15.6 (3.77)	15.9 (3.59)	14.6 (4.12)	
Median (Min, Max)	16.0 (0, 26.0)	16.0 (0, 25.0)	15.0 (4.00, 26.0)	
Missing	17 (5.1%)	11 (4.6%)	6 (6.7%)	
Sex				0.533
M	143 (43.1%)	107 (44.6%)	36 (40.0%)	
F	189 (56.9%)	133 (55.4%)	54 (60.0%)	
Age				0.261
Mean (SD)	61.2 (10.9)	61.6 (10.6)	60.1 (11.8)	
Median (Min, Max)	63.0 (31.0, 84.0)	64.0 (31.0, 83.0)	63.0 (31.0, 84.0)	
Age at PD onset				0.050
Mean (SD)	57.8 (10.9)	59.3 (11.0)	55.2 (10.6)	
Median (Min, Max)	60.0 (29.0, 80.0)	61.0 (32.0, 80.0)	58.5 (29.0, 70.0)	
Missing	211 (63.6%)	165 (68.8%)	46 (51.1%)	
PD duration (months)				0.028
Mean (SD)	37.3 (23.9)	33.4 (23.6)	43.2 (23.1)	
Median (Min, Max)	31.0 (2.92, 85.0)	29.0 (2.92, 80.1)	43.1 (4.01, 85.0)	
Missing	211 (63.6%)	165 (68.8%)	46 (51.1%)	
Antiparkinsonians				<0.001
No	198 (59.6%)	158 (65.8%)	40 (44.4%)	
Yes	134 (40.4%)	82 (34.2%)	50 (55.6%)	
Levodopa				<0.001
No	241 (72.6%)	188 (78.3%)	53 (58.9%)	
Yes	91 (27.4%)	52 (21.7%)	37 (41.1%)	
Dopamine agonists				0.002
No	274 (82.5%)	209 (87.1%)	65 (72.2%)	
Yes	58 (17.5%)	31 (12.9%)	25 (27.8%)	
MAO-B inhibitors				0.016
No	240 (72.3%)	183 (76.3%)	56 (62.2%)	
Yes	92 (27.7%)	57 (23.8%)	34 (37.8%)	
Entacapano				0.015
No	310 (93.4%)	230 (95.8%)	79 (87.8%)	
Yes	22 (6.63%)	10 (4.17%)	11 (12.2%)	
Levodopa equivalent daily dose				0.001
Mean (SD)	280 (714)	158 (323)	564 (1170)	
Median (Min, Max)	0 (0, 7630)	0 (0, 2400)	140 (0, 7630)	
Elixhauser comorbidities score				0.186
Mean (SD)	1.26 (3.14)	1.13 (2.85)	1.64 (3.83)	
Median (Min, Max)	0 (0, 20.0)	0 (0, 20.0)	0 (0, 19.0)	
MDS-UPDRS I-III				<0.001
Mean (SD)	18.4 (22.0)	14.4 (17.5)	29.5 (28.5)	
Median (Min, Max)	8.00 (0, 130)	7.00 (0, 106)	22.0 (0, 130)	
Missing	8 (2.4%)	2 (0.8%)	4 (4.4%)	
Dyskinesias				0.84
No	313 (96.3%)	230 (96.6%)	83 (95.4%)	
Yes	12 (3.69%)	8 (3.36%)	4 (4.60%)	
Missing	7 (2.1%)	2 (0.8%)	3 (3.3%)	
Motor fluctuations				<0.001
No	296 (91.1%)	226 (95.0%)	70 (80.5%)	
Yes	29 (8.92%)	12 (5.04%)	17 (19.5%)	
Missing	7 (2.1%)	2 (0.8%)	3 (3.3%)	
MoCA score				0.360
Mean (SD)	27.4 (2.76)	27.5 (2.57)	27.2 (3.24)	
Median (Min, Max)	28.0 (11.0, 30.0)	28.0 (11.0, 30.0)	28.0 (12.0, 30.0)	
Missing	19 (5.7%)	12 (5.0%)	7 (7.8%)	
Minimal cognitive impairment				0.999
No	148 (95.5%)	98 (95.1%)	50 (96.2%)	
Yes	7 (4.52%)	5 (4.85%)	2 (3.85%)	
Missing	177 (53.3%)	137 (57.1%)	38 (42.2%)	
PD Dementia				0.999
No	152 (98.1%)	102 (99.0%)	50 (96.2%)	
Yes	3 (1.94%)	1 (0.971%)	2 (3.85%)	
Missing	177 (53.3%)	137 (57.1%)	38 (42.2%)	

**Table 2:** SNPs in patients with probable REM-sleep behavior disorder (pRBD) or no RBD.

Number of Risk Alleles for Each SNP	Overall (N = 330)	No RBD (N = 240)	pRBD (N = 90)	Chi-sq *p*-Value	Additive Model OR (95% CI) *p*-Value, (AIC)	Dominant Model OR (95% CI) *p*-Value, (AIC)	Recessive Model OR (95% CI) *p*-Value, (AIC)	Full Model OR (95% CI) *p*-Value FDR *p*-Value
GBA_N370S_rs76763715				0.004	-	3.22 (1.48–7.07)	-	3.38 (1.45–7.93)
0	301 (91.3%)	226 (94.2%)	75 (83.3%)			0.002 (382.14)		0.004
1	29 (8.73%)	14 (5.83%)	15 (16.7%)			*SELECTED*		0.018
2	0 (0%)	0 (0%)	0 (0%)					
GBA_E365K_rs2230288				0.026	-	7.00 (1.47–49.51)	-	5.59 (1.05–42.09)
0	323 (97.9%)	238 (99.2%)	85 (94.4%)			0.02 (384.69)		0.050
1	7 (2.11%)	2 (0.833%)	5 (5.56%)			*SELECTED*		0.084
2	0 (0%)	0 (0%)	0 (0%)					
ACMSD.TMEM163_rs6430538				0.022	0.64 (0.45–0.89)	0.64 (0.39–1.06)	0.39 (0.19–0.77)	0.48 (0.22–0.99)
0	115 (34.9%)	77 (32.1%)	38 (42.2%)		0.01 (383.93)	0.08 (387.81)	0.009 (383.03)	0.050
1	142 (43.1%)	101 (42.1%)	41 (45.6%)				*SELECTED*	0.084
2	73 (22.0%)	62 (25.8%)	11 (12.2%)					
MCCC1_rs12637471				0.077	1.07 (0.69–1.64)	0.90 (0.54–1.48)	3.35 (0.98–11.92)	3.05 (0.83–11.65)
0	207 (62.3%)	149 (62.1%)	58 (64.4%)		0.74 (390.62)	0.69 (390.57)	0.05 (386.97)	0.110
1	112 (34.3%)	86 (35.8%)	26 (28.9%)				*SELECTED*	0.110
2	11 (3.31%)	5 (2.08%)	6 (6.67%)					
FAM47E.STBD1_rs6812193				0.077	1.22 (0.84–1.76)	1.02 (0.62–1.68)	2.14 (1.05–4.27)	1.77 (0.79–3.91)
0	132 (41.9%)	100 (41.7%)	37 (41.1%)		0.26 (389.49)	0.93 (390.72)	0.03 (386.31)	0.150
1	155 (46.7%)	118 (49.2%)	37 (41.1%)				*SELECTED*	0.170
2	38 (11.4%)	22 (9.17%)	16 (17.8%)					
SNCA_A53T_rs104893877				<0.001	-	7.37 (2.39–27.48)	-	8.21 (2.26–36.34)
0	312 (95.8%)	236 (98.3%)	80 (88.9%)			<0.001 (378.33)		0.002
1	14 (4.22%)	4 (1.67%)	10 (11.1%)			*SELECTED*		0.014
2	0 (0%)	0 (0%)	0 (0%)					
ANK2.CAMK2D_rs78738012				0.005	2.26 (1.34–3.82)	2.47 (1.39–4.36)	2.70 (0.32–22.81)	2.12 (1.08–4.10)
0	265 (80.1%)	203 (84.6%)	62 (68.9%)		0.002 (381.38)	0.001 (381.19)	0.32 (389.79)	0.030
1	61 (18.7%)	35 (14.6%)	26 (28.9%)			*SELECTED*		0.050
2	4 (1.20%)	2 (0.833%)	2 (2.22%)					
ZNF184_rs9468199				0.022	1.58 (1.04–2.41)	1.92 (1.17–3.16)	0.88 (0.19–3.04)	1.89 (1.08–3.33)
0	213 (64.8%)	165 (68.8%)	48 (53.3%)		0.03 (386.15)	0.009 (384.07)	0.85 (390.70)	0.030
1	105 (31.6%)	66 (27.5%)	39 (43.3%)			*SELECTED*		0.050
2	12 (3.61%)	9 (3.75%)	3 (3.33%)					
CTSB_rs1293298				0.108	0.96 (0.64–1.41)	0.77 (0.47–1.25)	1.87 (0.78–4.30)	1.91 (0.73–4.86)
0	157 (47.9%)	110 (45.8%)	47 (52.2%)		0.84 (390.69)	0.30 (389.66)	0.14 (388.67)	0.17
1	148 (44.6%)	115 (47.9%)	33 (36.7%)				*SELECTED*	0.17
2	25 (7.53%)	15 (6.25%)	10 (11.1%)					
COQ7.SYT17_rs11343				0.007	0.81 (0.57–1.15)	1.19 (0.69–2.10)	0.38 (0.17–0.75)	0.36 (0.15–0.78)
0	93 (28.0%)	70 (29.2%)	23 (25.6%)		0.25 (389.41)	0.51 (390.30)	0.009 (382.81)	0.010
1	168 (50.9%)	111 (46.3%)	57 (63.3%)				*SELECTED*	0.041
2	69 (21.1%)	59 (24.6%)	10 (11.1%)					
ZNF646.KAT8.BCKDK_rs14235				0.07	1.24 (0.87–1.75)	1.73 (1.03–3.01)	0.89 (0.45–1.69)	1.67 (0.93–3.08)
0	117 (35.5%)	93 (38.8%)	24 (26.7%)		0.22 (389.23)	0.04 (386.43)	0.74 (390.62)	0.09
1	158 (47.9%)	106 (44.2%)	52 (57.8%)			*SELECTED*		0.11
2	55 (16.6%)	41 (17.1%)	14 (15.6%)					

The Akaike Information Coefficient (AIC) was used to compare the additive, dominant, and recessive models. The one with the lowest AIC was selected for the multivariate model, provided the *p*-value was <0.15. FDR = False Discovery Rate *p*-value adjustment by the Benjamini and Hochberg step-up procedure.

**Table 3:** Performance of ML models for predicting possible RBD in the “validation” subsample.

Model	AUC ROC (%)	Sensitivity (%)	Specificity (%)	Calculated Prevalence (%)
Logistic regression	67.00	39 (20–61)	95 (87–99)	13 (7–21)
Bayes Naïve	67.00	39 (20–61)	95 (87–99)	13 (7–21)
Decision tree	55.27	13 (3–34)	97 (91–100)	5 (2–11)
Boosted Decision tree	79.12	22 (7–44)	99 (93–100)	6 (2–12)
Neural Network	80.63	35 (16–57)	95 (87–99)	12 (6–20)
SVM	76.00	9 (1–28)	99 (93–100)	3 (1–8)
Random Forrest	71.40	23 (15–32)	96 (89–99)	7 (3–14)

Sample size = 101 PD patients. Actual pRBD prevalence was 23% (15–32%).

**Table 4:** Odds ratios and performance of ML models, including clinical variables for predicting probable RBD in the “validation” subsample.

Logistic Regression Model	Only Genetic	Genetic + Clinical	Genetic + Clinical (LOOCV)
Model variables			
GBA_N370S_rs76763715 (Dominant)	1.67	1.20	1.62
GBA_E365K_rs2230288 (Dominant)	3.15	4.02	6.45
ACMSD.TMEM163_rs6430538 (Recessive)	0.44	0.57*	0.60
MCCC1_rs12637471 (Recessive)	3.31	2.48	2.34
FAM47E.STBD1_rs6812193 (Recessive)	1.91	1.59	1.62
SNCA_A53T_rs104893877 (Dominant)	6.49	3.32	3.65
ANK2.CAMK2D_rs78738012 (Dominant)	1.85	1.76	2.02
ZNF184_rs9468199 (Dominant)	1.85	2.01	2.12
CTSB_rs1293298 (Recessive)	2.07	2.25	2.19
COQ7.SYT17_rs11343 (Recessive)	0.40	0.38	0.34
ZNF646.KAT8.BCKDK_rs14235 (Dominant)	1.53	1.38	1.67
Levodopa-Equivalent Daily Dose	-	1.00	1.00
MDS-UPDRS I + II + III	-	1.02	1.02
Motor Fluctuations	-	0.89	1.06
Model parameters			
Sample Size	101	97	324
ROC (%)	67.0	82.5	78.3
Sensitivity (%)	39 (20–61)	48 (26–70)	38 (28–49)
Specificity (%)	95 (87–99)	97 (91–100)	95 (92–98)
Model parameters			
Sample Size	101	97	324
ROC (%)	67.0	82.5	78.3
Sensitivity (%)	39 (20–61)	48 (26–70)	38 (28–49)
Specificity (%)	95 (87–99)	97 (91–100)	95 (92–98)

## Data Availability

Data used in the preparation of this article were obtained from the Parkinson’s Progression Markers Initiative (PPMI) database (www.ppmi-info.org/data, accessed on 3 April 2023). For up-to-date information on the study, visit www.ppmi-info.org (accessed on 3 April 2023).
